# Longitudinal stability of fibromyalgia symptom clusters

**DOI:** 10.1186/s13075-018-1532-0

**Published:** 2018-02-27

**Authors:** Tanya L. Hoskin, Mary O. Whipple, Sanjeev Nanda, Ann Vincent

**Affiliations:** 1 Division of Biomedical Statistics and Informatics, Mayo Clinic, Rochester, MN USA; 2 Division of General Internal Medicine, Mayo Clinic, 200 First St SW, Rochester, MN 55905 USA; 3 School of Nursing, University of Minnesota, Minneapolis, MN USA

**Keywords:** Fibromyalgia, Longitudinal, Symptom cluster, Symptom stability

## Abstract

**Background:**

Using self-report questionnaires of key fibromyalgia symptom domains (pain, fatigue, sleep disturbance, function, stiffness, dyscognition, depression, and anxiety), we previously identified four unique symptom clusters. The purpose of this study was to examine the stability of fibromyalgia symptom clusters between baseline and 2-year follow-up.

**Methods:**

Women with a diagnosis of fibromyalgia completed the Brief Pain Inventory, Profile of Mood States, Medical Outcomes Study Sleep measure, Multidimensional Fatigue Inventory, Multiple Ability Self-Report Questionnaire, Revised Fibromyalgia Impact Questionnaire, and the 36-Item Short Form Survey Instrument at baseline. Follow-up measures were completed approximately 2 years later. The hierarchical agglomerative clustering algorithm previously developed was applied; agreement between baseline and follow-up was assessed with the κ statistic.

**Results:**

Among 433 participants, the mean age was 56 (range 20–85) years. The median Revised Fibromyalgia Impact Questionnaire total score was 57 (range 8–96). More than half of participants (58%) remained in the same cluster at follow-up as at baseline, which represented moderate agreement between baseline and follow-up (κ = 0.44, 95% confidence interval (CI) 0.37–0.50). Only two patients changed from high symptom intensity to low symptom intensity; similarly, only three moved from low to high.

**Conclusions:**

Fibromyalgia patients classified into four unique symptom clusters based on the key domains of pain, fatigue, sleep disturbance, function, stiffness, dyscognition, depression, and anxiety showed moderate stability in cluster assignment after 2 years. Few patients moved between the two extremes of severity, and it was slightly more common to move to a lower symptom level than to worsen.

**Trial registration:**

Not applicable.

## Background

Chronic widespread pain, stiffness, fatigue, sleep disturbance, cognitive difficulties, and mood disturbance are considered core symptoms of fibromyalgia. In 2008, the Outcome Measures in Rheumatology fibromyalgia working group recommended evaluation of these core symptoms in addition to measures of function and biomarkers, when available, in all clinical research into fibromyalgia [1]. In a previous study [2], we identified four unique symptom clusters using self-report questionnaires in a sample of 581 women with fibromyalgia using these core Outcome Measures in Rheumatology symptom domains. These clusters included: 1) a low symptom intensity group; 2) a moderate symptom intensity group with low anxiety and depression; 3) a moderate symptom intensity group with higher anxiety and depression; and 4) a high symptom intensity group.

Our results are consistent with previous studies that have also reported the presence of symptom subgroups, or *clusters,* in samples of patients with fibromyalgia [3–7]. For example, Yim and colleagues [7] identified four clusters based on physical and psychological assessments and measures of pain: 1) high pain and physical and mental impairment, with low social support; 2) moderate pain and physical impairment, with mild mental impairment and moderate social support; 3) moderate pain and mental impairment, with low physical impairment and social support; and 4) low pain, with normal physical and mental function and high social support. Similarly, Docampo and colleagues [6] identified three clusters on the basis of fibromyalgia symptoms and comorbid conditions: 1) low symptoms and comorbid conditions; 2) high symptoms and comorbid conditions; and 3) high symptoms but low comorbid conditions. These and previous studies suggest the presence of distinct symptom subgroups within samples of patients with fibromyalgia. There is usually one subgroup with high physical and/or psychological symptoms, one subgroup with low physical and psychological symptoms, and one or more moderate symptom subgroup(s). The presence of distinct symptom subgroups argues against uniform assessment and treatment of fibromyalgia and supports the need for further research to identify symptom patterns and enhance symptom management.

Existing research examining the longitudinal stability of individual fibromyalgia symptoms suggests that changes in individual symptoms in patients with fibromyalgia are generally small and of doubtful clinical significance [8]. No study to date has examined changes in a patient’s entire symptom profile over time. Therefore, the purpose of this study was to examine the stability of individual patient profiles (symptom clusters) over a 2-year period in a well-characterized cohort of patients with fibromyalgia.

## Methods

The Mayo Clinic Institutional Review Board approved this study. All participants provided written informed consent.

### Sample

Participants for this study were women who met Fibromyalgia Research Criteria [9] at baseline and participated in a previously published cluster analysis of fibromyalgia symptoms [2].

### Data collection

Participants were invited to complete follow-up questionnaires by mail. These included the Brief Pain Inventory (BPI) [10], the 30-item Profile of Mood States (POMS) [11], the Medical Outcomes Study Sleep measure (MOS-Sleep) [12], the Multidimensional Fatigue Inventory (MFI) [13], the Multiple Ability Self-Report Questionnaire (MASQ) [14], the Revised Fibromyalgia Impact Questionnaire (FIQR) [15], and the 36-Item Short Form Survey Instrument (SF-36; RAND) [16]. All instruments included have been used extensively in fibromyalgia research [17] and have been described in detail in the original cluster analysis [18].

### Measures

#### Brief Pain Inventory

The BPI is a validated self-report measure of pain [10] that yields two subscales: pain severity and pain interference. Scores range from 0 to 10, with higher scores indicating greater pain. The BPI has been used extensively in fibromyalgia studies [17, 19–22]. The pain severity subscale was used to represent the symptom domain of pain.

#### Profile of Mood States

The POMS is a validated self-report measure of mood [11] that is composed of six subscales: 1) depression-dejection, 2) tension-anxiety, 3) fatigue-inertia, 4) vigor-activity, 5) anger-hostility, and 6) confusion-bewilderment. Scores range from 0 to 20, with higher scores indicating worse mood, except for the vigor-activity scale [23–25]. The depression-dejection and tension-anxiety subscales were used to represent the symptom domains of depression and anxiety.

#### Medical Outcomes Study Sleep measure

The MOS-Sleep is a validated self-report measure of sleep that results in two summary scores: the Sleep Problems Index I (six items) and the Sleep Problems Index II (nine items) [12]. Scores range from 0 to 100, with higher scores indicating poorer sleep. This instrument has been used extensively in fibromyalgia studies [26–28]. The Sleep Problems Index II was used to represent the symptom domain of sleep.

#### Multidimensional Fatigue Inventory

The MFI is a validated self-report measure of fatigue [13]. Subscale scores range from 4 to 20, with higher scores indicating greater fatigue. The MFI has been used extensively in fibromyalgia research [20, 29, 30]. The MFI Physical Fatigue subscale was used to represent the symptom domain of fatigue.

#### Multiple Ability Self-Report Questionnaire

The MASQ is a self-report measure of cognition [14]. Total scores range from 0 to 190, with higher scores indicating greater perceived difficulties with cognition. The MASQ has been used extensively in fibromyalgia research [31–33]. We used the MASQ total score to represent the symptom domain of dyscognition.

#### Revised Fibromyalgia Impact Questionnaire

The FIQR is a validated self-report measure that assesses the symptoms, physical functioning, and overall impact of fibromyalgia [15]. Scores range from 0 to 100, with higher scores indicating greater symptom burden. It is the most commonly used outcome measure in fibromyalgia clinical trials [17, 26, 34, 35]. For this analysis, we selected the FIQR stiffness question to represent the symptom domain of stiffness.

#### 36-Item Short Form Survey Instrument

The SF-36 version 2 is a 36-item, validated self-report measure that assesses disease burden [16]. It consists of eight subscales and two summary scores (physical and mental components). Component scores range from 0 to 100, with higher scores indicating better health. The SF-36 has been widely used in fibromyalgia clinical trials [36–38].

### Statistical analysis

Using classification rules derived from the prior hierarchical agglomerative cluster analysis performed in these patients [2] with their measures at study enrollment, we classified each participant into one of four clusters using the existing algorithm on their measures recorded at follow-up. The agreement between cluster assignment at baseline and follow-up was assessed with the κ statistic and 95% confidence intervals (CIs). Agreement between the two time points on individual instrument scores was assessed with the intraclass correlation coefficient, and changes in scores were assessed with paired *t* tests. The percentage change in measures was calculated as ((follow-up score – baseline score)/baseline score) × 100 for further descriptive analysis. A change of 30% or more from baseline was considered clinically important [39], and the proportion meeting this criterion was reported for each instrument. All tests were two-sided, with an α level of 0.05 used to determine statistical significance. Analysis was performed using JMP (Version 10; SAS Institute Inc.).

## Results

From the original sample of 581 patients, 433 (74.5%) completed the follow-up survey and had complete data on all clustering variables. Demographic characteristics of the sample with complete data are shown in Table 1. The median age was 56 years, and the median body mass index was 29 kg/m^2^. The median total FIQR score was 57, and the majority of patients (83.8%; *n* = 363) continued to meet Fibromyalgia Research Survey criteria for the diagnosis of fibromyalgia at follow-up.

**Table 1:** Patient characteristics at baseline (*N* = 433)

Characteristic	Value^a^
Age, years	56 (20–85)
BMI, kg/m^2^	29 (13–58)
BMI category	(*n* = 431)
< 25 kg/m^2^	114 (26)
25–29.99 kg/m^2^	125 (29)
30–34.99 kg/m^2^	98 (23)
≥ 35 kg/m^2^	94 (22)
FIQR total score	57 (8–96)
SF-36 physical composite score	30 (8–53)
SF-36 mental composite score	41 (7–67)
FM Research Survey Criteria, WPI	13 (3–19)
FM Research Survey Criteria, SS	9 (5–12)

*BMI* body mass index, *FIQR* Revised Fibromyalgia Impact Questionnaire, *FM* fibromyalgia, *SF-36* 36-Item Short Form Survey Instrument, *SS* symptom severity score, *WPI* widespread pain index

^a^Values are median (range) or number of patients (%)

Concordance between cluster membership at baseline and follow-up is shown in Table 2. Overall, 251 participants (58%) remained in their baseline cluster at follow-up, for a κ statistic of 0.44 (95% CI 0.37–0.50), which suggests moderate agreement or stability between baseline and 2-year follow-up. The highest concordance was observed for participants originally classified in cluster 1 and cluster 4, for which 68% and 70%, respectively, remained in the same cluster at follow-up. Baseline cluster 2 and 3 patients showed more reclassification, with only 53% and 43%, respectively, in the same cluster at follow-up. Baseline cluster 2 patients were most commonly reclassified into cluster 1 (21%), followed by cluster 3 (19%). Baseline cluster 3 patients were also most commonly reclassified into cluster 1 (32%). Patients at the extremes of severity (cluster 1 low severity, and cluster 4 high severity) were rarely reclassified in the opposite extreme at follow-up: three baseline cluster 1 patients (3%) and two baseline cluster 4 patients (2%).

**Table 2:** Agreement between baseline cluster assignment and follow-up cluster assignment

	Follow-up cluster^a^				
Baseline cluster	1	2	3	4	*n*
1	**79 (68)**	21 (18)	13 (11)	3 (3)	116
2	27 (21)	**69 (53)**	25 (19)	10 (8)	131
3	32 (32)	11 (11)	**43 (43)**	14 (14)	100
4	2 (2)	9 (10)	15 (17)	**60 (70)**	86
*n*	140	110	96	87	433

^a^Values are number of patients (%)

Bold entries indicate the patients for whom cluster did not change during follow-up

Clusters are as follows: 1, generally low symptom severity; 2, moderate symptom severity but low anxiety and depression; 3, moderate symptom severity with higher anxiety and depression; 4, generally high symptom severity

When evaluating the stability of individual symptoms, intraclass correlation coefficient values showed generally good consistency between the two time points for individual symptom domain measures, with values of 0.60 or greater for all scales except FIQR stiffness, which had an intraclass correlation coefficient of only 0.23 (Table 3). Small but statistically significant improvements were observed at follow-up for the symptoms of fatigue, stiffness, and dyscognition. The FIQR total score and SF-36 physical composite score also improved significantly by means of 1.50 points (*P* = 0.02) and 0.76 points (*P* = 0.03), respectively. Significant worsening was not observed for any scale.

**Table 3:** Consistency and degree of change between baseline and follow-up for individual measures

		Mean (SD) value		Mean (95% CI) paired difference	Number of patients (%)		
Measure	ICC	Baseline	Follow-up		Worsened by ≥ 30%	Within 30% of baseline at follow-up	Improved by ≥ 30%
Cluster analysis measures							
MFI	0.61	15.7 (3.6)	15.2 (3.7)	−0.54 (−0.85, −0.23)	44 (10)	346 (80)	43 (10)
MOS-Sleep	0.66	55.2 (18.9)	55.4 (19.1)	0.20 (−1.29, 1.69)	77 (18)	304 (70)	52 (12)
BPI severity	0.62	5.1 (1.8)	5.0 (1.8)	−0.11 (−0.26, 0.03)	79 (18)	285 (66)	69 (16)
FIQR function	0.72	14.0 (7.2)	13.7 (7.4)	−0.30 (−0.82, 0.22)	106 (24)	226 (52)	97 (22)
FIQR stiffness	0.23	7.2 (2.2)	6.6 (2.4)	−0.62 (−0.85, −0.40)	53 (12)	287 (66)	90 (21)
MASQ	0.80	95.1 (21.8)	93.7 (22.3)	−1.4 (−2.7, −0.05)	19 (4)	405 (94)	9 (2)
POMS depression-dejection	0.61	6.6 (5.0)	6.3 (4.9)	−0.25 (−0.67, 0.16)	128 (30)	172 (40)	133 (31)
POMS tension-anxiety	0.60	7.0 (4.6)	7.2 (4.7)	0.15 (−0.24, 0.55)	131 (30)	198 (46)	104 (24)
Global measures not included in cluster analysis							
FIQR total (*n* = 410)	0.75	55.4 (19.0)	53.9 (19.9)	−1.50 (−2.84, −0.17)	59 (14)	300 (73)	51 (12)
SF-36 physical (*n* = 380)	0.69	30.3 (8.7)	31.0 (8.6)	0.76 (0.07, 1.44)	19 (5)	305 (80)	56 (15)
SF-36 mental (n = 380)	0.60	40.6 (12.3)	40.2 (12.7)	−0.37 (−1.50, 0.75)	47 (12)	267 (70)	66 (17)
FM Research Survey Criteria, WPI	0.47	12.6 (3.8)	12.1 (4.0)	−0.58 (−0.20, −0.95)	75 (17)	272 (63)	86 (20)
FM Research Survey Criteria, SS	0.57	8.8 (2.0)	8.2 (2.3)	−0.63 (−0.43, −0.81)	31 (7)	344 (80)	57 (13)

*BPI* Brief Pain Inventory, *CI* confidence interval, *FIQR* Revised Fibromyalgia Impact Questionnaire, *FM* fibromyalgia, *ICC* intraclass correlation coefficient, *MASQ* Multiple Ability Self-Report Questionnaire, *MFI* Multidimensional Fatigue Inventory, *MOS-Sleep* Medical Outcomes Study Sleep measure, *POMS* Profile of Mood States, *SD* standard deviation, *SF-36* 36-Item Short Form Survey Instrument, *SS* symptom severity score, *WPI* widespread pain index

Although statistically significant changes between the two time points were observed for some instruments, there were few clinically meaningful differences, with most patients remaining within 30% of baseline at follow-up for all instruments except POMS depression-dejection and POMS tension-anxiety (Table 3). On the depression-dejection and tension-anxiety scales, more than half of patients changed by more than 30% from baseline to follow-up, but those changes reflected both improvements (31% and 24%, respectively, improving by ≥ 30%) and worsening (30% worsening by ≥ 30% for each instrument). On global measures, 70% or more of patients remained within 30% of baseline: 73% for FIQR, 80% for SF-36 physical, and 70% for SF-36 mental.

For patients who moved to a better cluster (i.e., a lower symptom burden profile) between baseline and follow-up, the symptom domains demonstrating the largest changes were depression (mean 26% decrease from baseline), stiffness (mean 23% decrease from baseline), and function (mean 21% decrease from baseline) (Table 4). Statistically significant changes were observed for all symptoms except sleep. Similarly, for patients who moved to a worse cluster, all symptoms showed a statistically significant increase. Among those who were in a worse cluster at follow-up, the symptom domains demonstrating the largest changes were depression (mean 112% increase from baseline), anxiety (mean 102% increase from baseline), and function (mean 58% increase in score, indicating decreasing function, from baseline).

**Table 4:** Percentage change on cluster analysis measures: movement to a better cluster or worse cluster^a^

Measure	Better cluster at follow-up(*n* = 96)	Worse cluster at follow-up(*n* = 86)
MFI	−12.42 (−16.96, −7.89)	9.92 (4.73, 15.10)
MOS-Sleep	−3.72 (−12.57, 5.13)	25.46 (13.36, 37.56)
BPI	−15.62 (−21.81, −9.44)	35.28 (16.01, 54.54)
FIQR function	−21.36 (−30.70, −12.02)	58.38 (24.45, 92.30)
FIQR stiffness	−23.26 (−32.24, −14.28)	31.27 (14.03, 48.52)
MASQ	−5.85 (−8.28, −3.43)	8.59 (5.07, 12.10)
POMS depression-dejection	−26.28 (−39.67, −12.88)	111.95 (73.43, 150.48)
POMS tension-anxiety	−15.97 (−29.11, −2.83)	101.90 (62.73, 141.06)

*BPI* Brief Pain Inventory, *FIQR* Revised Fibromyalgia Impact Questionnaire, *MASQ* Multiple Ability Self-Report Questionnaire, *MFI* Multidimensional Fatigue Inventory, *MOS-Sleep* Medical Outcomes Study Sleep measure, *POMS* Profile of Mood States

^a^Values are mean (95% confidence interval) percentage change from baseline to follow-up

## Discussion

The results of our study indicate relative stability in fibromyalgia symptom severity and symptom clusters from baseline to 2-year follow-up. This cluster stability was most apparent in two of the four subgroups: those with the lowest level of symptoms (cluster 1) and those with the highest level of symptoms (cluster 4). In contrast, patients in clusters 2 and 3 with moderate symptom severity demonstrated greater fluctuation. Patients who shifted to a different cluster at follow-up were slightly more likely to move to a lower severity cluster than to a higher one.

Although the clinical implications of these findings are not fully understood, our results suggest that most patients with fibromyalgia generally did not have progressive worsening and maintained their baseline symptom severity profile. This information provides the opportunity for further research evaluating individualized management tailored to each patient’s multisymptom profile. Fibromyalgia management remains challenging for clinicians despite the availability of several pharmacologic and nonpharmacologic therapies [40]. This challenge may be partly due to the heterogeneity of the syndrome and a one-size-fits-all approach that is currently used for disease management in patients of varying symptom severity patterns. Improved methods of clinical stratification to categorize symptom severity across all relevant domains at the time of diagnosis could improve clinical management of fibromyalgia and warrants further study.

Patients in the most severe cluster (cluster 4) and the lowest symptom severity cluster (cluster 1) showed the greatest stability, with 70% and 68%, respectively, remaining in the same cluster at follow-up. Given their overall low symptom severity, cluster 1 patients may be excellent candidates for nonpharmacologic treatments (e.g., aerobic and strength training, cognitive behavioral therapy) as currently recommended by the European League Against Rheumatism [41]. In contrast, cluster 4 patients, whose symptoms remained severe over time for the most part, may be best served by early referral to a multidisciplinary pain clinic as recommended by the European League Against Rheumatism [41], rather than treating patients in the primary care setting or referring them to multiple subspecialists. In addition, patients with a high degree of anxiety and depression may also benefit from early referral to mental health specialists.

Of interest, among patients who moved to a worse cluster, the symptoms that drove the change were predominantly depression and anxiety. This is not surprising since previous studies report the detrimental effects of comorbid depression and anxiety on overall symptom severity [42, 43] and because of the bidirectional effect of depression in samples of patients with fibromyalgia [44]. Research has also demonstrated that patients with fibromyalgia with comorbid mood disorders and anxiety tend to have poorer outcomes than patients with predominantly physical and lower psychological symptoms [42]. Our finding that mood and anxiety tended to drive changes in cluster categorization highlights the importance of regular mood and anxiety assessment and referral to mental health specialists, as appropriate, in patients with fibromyalgia who have worsening symptoms.

Our approach using multiple questionnaires is not suited for routine clinical care. It has been suggested that the stratification of fibromyalgia severity in busy clinical practices could be accomplished through the use of single instruments [45]. For example, the visual analogue scales of the FIQR, the complete FIQR (although this involves more complex scoring), or the Fibromyalgia Symptom Score could be used to rapidly gauge both severity and constellation of fibromyalgia symptoms [45].

The current study has several limitations. One key limitation was the lack of data regarding medications and other treatment modalities that patients may have used during the 2-year time frame; however, it was not feasible to collect information at the level of detail required, and patients’ recall of medications and changes in medication regimen are frequently inaccurate [46]. A second limitation is the loss of approximately 25% of our sample at follow-up, which may have biased our results. However, patients without follow-up did not differ significantly in baseline cluster distribution from those who did complete follow-up (data not shown). Therefore, it is unlikely that this influenced our results. Another major limitation is the lack of data on work/disability status, socioeconomic status, and medical and psychiatric comorbid conditions. These data were not collected as part of this mailed survey to limit the participant burden associated with the inclusion of more than 200 questionnaire items. In our previous cross-sectional study [2], the percentage of patients on work disability differed significantly between clusters, with the highest percentage of patients on disability in cluster 4 and the lowest percentage in cluster 1. In addition, because of our inclusion of only women with fibromyalgia identified through a clinical registry [47], we cannot comment on the generalizability of our results to community samples, men, or adolescents with fibromyalgia.

## Conclusions

The results of our study suggest that fibromyalgia symptom severity and symptom patterns do not change substantially over 2 years in patients with low and high symptom intensity but are less stable in patients with moderate symptom intensity. This may have important clinical implications, particularly for patients with high symptom severity who may be more likely to benefit from early identification and timely implementation of evidence-based modalities. Our study also suggests that in patients with fibromyalgia with low symptom severity, the disorder may be relatively stable over 2 years and does not necessarily progress in severity. These findings support future studies of categorization of patients’ symptom severity at initial evaluation to appropriately guide disease management.

## Abbreviations

BPI: Brief Pain Inventory
CI: Confidence interval
FIQR: Revised Fibromyalgia Impact Questionnaire
MASQ: Multiple Ability Self-Report Questionnaire
MFI: Multidimensional Fatigue Inventory
MOS-Sleep: Medical Outcomes Study Sleep measure
POMS: Profile of Mood States
SF-36: 36-Item Short Form Survey Instrument
